# Developmental studies in fragile X syndrome

**DOI:** 10.1186/s11689-020-09310-9

**Published:** 2020-05-02

**Authors:** Khaleel A. Razak, Kelli C. Dominick, Craig A. Erickson

**Affiliations:** 1 Department of Psychology and Graduate Neuroscience Program, University of California, Riverside, USA; 2 Department of Psychiatry and Behavioral Neuroscience, University of Cincinnati, Cincinnati, OH USA; 3 Division of Child and Adolescent Psychiatry, Cincinnati Children’s Hospital Medical Center, 3333 Burnet Avenue MLC 4002, Cincinnati, OH 45229 USA

## Abstract

Fragile X syndrome (FXS) is the most common single gene cause of autism and intellectual disabilities. Humans with FXS exhibit increased anxiety, sensory hypersensitivity, seizures, repetitive behaviors, cognitive inflexibility, and social behavioral impairments. The main purpose of this review is to summarize developmental studies of FXS in humans and in the mouse model, the *Fmr1* knockout mouse. The literature presents considerable evidence that a number of early developmental deficits can be identified and that these early deficits chart a course of altered developmental experience leading to symptoms well characterized in adolescents and adults. Nevertheless, a number of critical issues remain unclear or untested regarding the development of symptomology and underlying mechanisms. First, what is the role of FMRP, the protein product of *Fmr1* gene, during different developmental ages? Does the absence of FMRP during early development lead to irreversible changes, or could reintroduction of FMRP or therapeutics aimed at FMRP-interacting proteins/pathways hold promise when provided in adults? These questions have implications for clinical trial designs in terms of optimal treatment windows, but few studies have systematically addressed these issues in preclinical and clinical work. Published studies also point to complex trajectories of symptom development, leading to the conclusion that single developmental time point studies are unlikely to disambiguate effects of genetic mutation from effects of altered developmental experience and compensatory plasticity. We conclude by suggesting a number of experiments needed to address these major gaps in the field.

## Introduction

Fragile X syndrome (FXS) is the most common inherited cause of intellectual disability and most common single gene cause of autism. Common symptoms of FXS include hyperactivity, repetitive behaviors and cognitive inflexibility, hypersensitivity to sensory stimuli and seizures, social and language impairments, intellectual disability, and increased anxiety. FXS occurs as a result of *Fmr1* gene hypermethylation due to an unstable CGG triplet repeat expansion (> 200 repeats) in the 5′ untranslated region of the *Fmr1* gene located on the X chromosome, which leads to gene methylation, inactivation, and resultant loss of fragile X mental retardation protein expression (FMRP; [147]). FMRP functions as a translational regulator, affecting synthesis of many proteins including those involved in synaptic pruning during development. Additional functions such as modulation of ion channels have also been proposed [15, 29, 39]. A recent meta-analysis estimates the frequencies of individuals with the full mutation FXS allele to be approximately 1 in 7000 males and 1 in 11,000 females [75]. As an X-linked disorder, the phenotypic expression is quite variable in females because of random inactivation and potential compensation by the normal X chromosome [108]. Therefore, the prevalence of females who carry the full mutation allele and display the phenotypic features of FXS is less than 1 in 11,000 [75].

The *Fmr1* knockout (KO) mouse has been the most widely studied animal model of FXS, but other model systems such as drosophila and *Fmr1* KO rats have also contributed valuable information regarding basic biological functions of FMRP and mechanisms of pathophysiology when FMRP is removed. Over the past two decades, considerable progress has been made in terms of our understanding of symptomology and underlying neurobiology in humans with FXS and animal models. Because of the monogenic origin of the disorder and significant overlap with autism, the interest in FXS is at least partly due to the potential for discoveries about FXS to serve as a paradigm for studies of neurodevelopmental disorders more broadly.

While several potential therapeutics show efficacy in reversing symptoms in the *Fmr1* KO mice, they have failed clinical trials [12]. A number of reasons may explain these failures including the validity of the preclinical models, validity of outcome measures, insufficient stratification of patients, optimality of dose and duration, and finally, appropriate treatment age. To address optimal treatment ages, we focused on developmental studies of both humans and mice to generate hypotheses based on published data. FXS is a neurodevelopmental disorder. However, there is little understanding of whether and how functions of FMRP change during development. Only a few studies have systematically examined the roles of this protein for normal brain development over time. The developmental trajectories of symptoms and their relationship to changes in underlying mechanisms are only beginning to be understood. In human work, only a handful of studies have examined early childhood development (age < 5). Only a few longitudinal studies with large sample sizes have examined trajectories of developmental change in FXS phenotypes. There is little data-driven guidance on optimal time points in development for clinical tests of potential therapeutics. While studies point to early detection of many phenotypes, whether developmental treatment has longer lasting benefits than adult treatments remains unknown. In the following sections, we summarize the animal and human studies that have bearing on these issues and suggest a number of future experimental approaches that are needed to fill major gaps in our knowledge of FXS development.

## Animal Models

### Development of mechanisms and phenotypes in animal models of FXS

There is an extensive literature on preclinical animal models of FXS, mostly in mice. Many of these studies have examined phenotypes during early development either due to convenience of methodology (e.g., in vitro slices are easier to record from in very young mice) or to examine developmental trajectory. Almost all of the developmental trajectory studies in animal models use a cross-sectional approach. Such studies conducted before 2014 have been comprehensively reviewed elsewhere [24, 102]. Here, we summarize key studies done in the past few years and focus on the gaps and paradoxes in the developmental literature on animal models. The major point made by these studies is that many of the phenotypic differences between KO and WT mice are developmentally transient, suggesting the importance of studying trajectories. This is all the more relevant if altered brain responses attributed to an imbalance in excitatory and inhibitory function reflects compensatory plasticity, and is not necessarily the primary defect driven by the genetic mutation causing neurodevelopmental disorders [4, 102, 136].

### Developmental regulation of FMRP expression

FMRP levels are developmentally regulated [46] in the mouse brain. Peak FMRP expression during development occurs between P3 and P12 in different brain regions and is reduced in adulthood. This age coincides with the onset of sensory stimulation and developmental critical period plasticity windows, suggesting an important role for FMRP in experience-dependent plasticity. In the auditory and somatosensory cortex, peak FMRP levels are seen between P7 and P12. In the hippocampus, cerebellum, striatum, and brainstem, the peak FMRP expression is between P3 and P7. FMRP is expressed across cell types in multiple brain regions in adults. Whether different cell types in these regions show different developmental profiles of FMRP expression has not been studied.

Such consistently high levels of FMRP expression in early development suggest that the function of FMRP is essential during this time period. FMRP regulates protein translation by association with a vast array of mRNAs [16, 148]. A well-known phenotype in the *Fmr1* KO mouse brain is increased protein synthesis [93, 109]. However, it remains unclear if this phenotype is observed across development and if different brain regions have similar changes in protein synthesis across development. Most studies examine a single or a few time points in specific brain regions. This issue is further complicated in that different cell types may show different proteins whose levels are increased or decreased. Recent studies of protein synthesis in humans with FXS show that at least some of the patients show decreased protein synthesis [81, 114], opposite to that seen in the mouse model. While this naturally leads to questions on the utility of the mouse model, this paradox can be potentially resolved if developmental profiles of protein synthesis in FXS in different brain regions are characterized in rodent models, and if possible, in humans. This remains an outstanding issue.

When adult phenotypes of a genetic disorder are considered, it is difficult to disentangle direct effects of the genetic mutation from effects of altered developmental experience [4]. One approach is to study mechanisms of pathophysiology early in development before the animal has had considerable time for developmental experience to shape the brain. Such studies show that the lack of FMRP leads to several synaptic, circuit-level and behavioral alterations, many of which are seen very early in development [102]. These studies show that FMRP is clearly involved in brain development, particularly in shaping synaptic connectivity. These deficits most likely impact experience dependent plasticity and lead to permanent changes in brain and behavior. However, in most studies of potential therapeutics, treatment of adult mice or brains shows reversal of phenotypes. This would suggest that the structural and functional brain alterations that develop due to the lack of FMRP in early development could be fixed by focusing on specific molecular pathways in adulthood. It is worth highlighting this as a paradox. Given the number of mRNAs under the control of FMRP, the high expression of FMRP during critical periods of brain growth, and extensive evidence that altered activity early in development causes long-lasting and potentially irreversible changes into adulthood, the ability of adult treatment to reduce a vast range of symptoms in animal models is quite remarkable. This ability of adult treatment to reverse symptoms has major implications for whether the *Fmr1* gene can be reactivated in adults and what the prognosis might be. However, we argue here that two major classes of experiments are needed to fully support the idea that adult treatment alone may be sufficient to treat symptoms of FXS. We also point to the scarcity of such studies in the literature.

1. Test the necessity and sufficiency of FMRP in normal brain development. These experiments, performed in animal models, will entail removing FMRP during an early developmental window, and re-expressing it later in adults. The reverse experiment in which FMRP is removed only in adulthood will address the sufficiency question. In both cases, adult phenotypes should be examined to determine if early normal FMRP is necessary and sufficient to reduce symptoms in adults.
2. To be performed first in animal models, experiments should determine the longevity of benefits in terms of symptoms if early developmental versus late (adult) treatment windows are used. Depending on the results in preclinical studies, clinical trial designs should explore this issue in children.

Currently, only a few studies in the literature have performed the experiments to test the role of FMRP in development, and they point to different outcomes. In the drosophila model of FXS (*dFmr1* KO flies), the small ventrolateral neurons (sLNv) are GABA-responsive, PDF-peptide expressing neurons involved in circadian pattern generation [44]. *dFmr1* KO flies do not exhibit circadian rhythmicity. sLNv in *dFmr1* KO flies have overgrowth and overextension of synaptic arbor. Overexpression of *dFmr1* specifically in these neurons can reduce synaptic arbor. The expression of *dFmr1* in early development (time of neurogenesis and migration) or in adults does not change synaptic architecture. However, *dFmr1* expression during an intermediate developmental window (pupal days 3, 4) normalize synaptic arbor. This is also the time window when dFMRP levels are normally high in WT flies. While the effects of normalizing synaptic arbor with this brief period of *dFmr1* expression on circadian patterns were not reported, this study shows the sufficiency of normal sLNv *dFmr1* levels during a developmental window in normal synaptic development. Doll et al. [31] also concluded that FMRP function during early developmental critical window is absolutely required for drosophila olfactory circuit organization.

A more recent study in the mouse shows that FMRP in early development is not necessary for the normal expression of a prefrontal cortex (PFC) dependent task, the trace eye-blink conditioning in adult mice [127]. Mice were trained to associate a sound with a puff of air to the eyelid. Eye blinks could be elicited with the sound alone once training is complete. This is called a “trace” conditioning paradigm because of the temporal gap between sound cue and puff of air; the gap makes this task PFC dependent. FMRP deletion only in the adult revealed an increase in the proportion of non-learners and a delay in the onset of learning. This was true in both the full *Fmr1* KO mice and the conditional KO mice in which FMRP was specifically removed from the PFC. Mice without FMRP in PFC in early development, but normal FMRP expression in PFC as adults, did not show learning deficits. This indicates acute roles for FMRP in adult PFC in acquisition and maintenance of eye-blink trace conditioning. Arsenault et al. [5] used viral vectors to express FMRP with the synapsin promoter (for expression in neurons) in mice and determined behavioral outcomes in adults. A single intracerebroventricular injection of adeno-associated viral vector was made in P0–P2 mouse pups. FMRP expression in adult brains was mostly seen in the forebrain. When adult behaviors were examined, hyperactivity, elevated plus, and acoustic startle abnormalities were significantly rescued. While the developmental trajectory of FMRP expression levels is unclear from this study, the results point to the potential utility of post-natal expression of FMRP in reversing symptoms of FXS. Zeier et al. [149] expressed FMRP bilaterally in the hippocampus by injecting the viral vector construct in 5-week-old mice (adolescence/young adult age). They observed that hippocampal enhanced LTD phenotype is rescued with FMRP expression in the adult hippocampus. This further suggests that FMRP expression in adults is sufficient to restore normal plasticity in hippocampus.

While the mouse and drosophila studies come to different conclusions regarding the necessity of FMRP in early development, a number of differences (even beyond species differences) need to be considered. Terms borrowed from developmental neuroendocrinology may be applicable here to provide a scaffold for future investigations of FMRP in neurobehavioral functions. In the drosophila study, the outcome measured was an “organizational” role of FMRP in terms of synaptic arbor. Whereas in the mouse eye-blink conditioning study, the outcome measured was an “activation” effect of FMRP in terms of experience dependent plasticity. These studies suggest that depending on the outcome measure, the necessity and sufficiency of FMRP in development of FXS symptomology may be different. Perhaps, FMRP plays an early role in synaptic organization during the time of high expression in early development (P3–P12 in mice). The levels decrease into adulthood during which FMRP is necessary for activation of experience dependent plasticity. These results have major implications for the outcome measures that need to be considered in adolescent and adult treatments of patients. Mechanisms and biomarkers that are involved with organizational effects may be irreversibly altered if adult treatment is utilized, while activation effects involved in learning outcomes may be corrected.

### Lessons from Angelman and Rett syndromes

The effectiveness of developmental treatment in providing long-lasting benefits has been shown in other disorders such as developmental hearing loss [86] and Angelman syndrome. Particularly pertinent to this review is Angelman syndrome (AS), a neurodevelopmental disorder that has a number of features on the autism spectrum. AS results from the loss of function of maternal UBE3A allele. The pharmacological reactivation of the paternal Ube3a gene in adult mice was not effective in reducing most symptoms of the disorder including epilepsy. However, a systematic examination of critical developmental windows for reactivation of Ube3a in mice revealed that anxiety, repetitive behaviors, and epilepsy were normalized only if reactivation is done from embryonic development [128]. Motor behaviors are normalized if reactivation is commenced at ~P21, but not in adults. Synaptic plasticity (long-term potentiation) in the hippocampus is normal if Ube3a is activated at any time. This is consistent with the *Fmr1* KO mouse study in which even adult expression of *Fmr1* is sufficient for learning related behaviors (“activational effects”). In a recent paper on an AS mouse model, Gu et al. [53] identified an abnormal accumulation of perineuronal nets in the hippocampus during epileptogenesis and showed that reinstatement of Ube3a from ~P21 reduces PNN and epileptogenesis in the AS model mice, but adult reinstatement did not. Together, these results suggest distinct sensitive windows during which Ube3a reactivation has to occur for different phenotypes. This is in contrast to another neurodevelopmental condition, Rett syndrome, in which both developmental and adult activation of Mecp2 (mutation in the X-linked MECP2 gene causes Rett syndrome) can successfully reverse Rett syndrome associated pathologies in a mouse model [55]. These studies highlight the importance of understanding functions of Ube3a, Mecp2, and Fmr1 at different developmental ages.

However, the topic of critical windows for treatment or reactivation efficacy has received very little attention in FXS. Only a few studies have directly addressed the efficacy of developmental versus adult treatment in reversing phenotypes. Dansie et al. [27] showed that minocycline treatment in *Fmr1* KO mice was effective in reversing anxiety-like behaviors. When adult mice were treated, a continuous administration of the drug was needed to maintain the effects. However, treatment in young mice has lasting effects after treatment cessation. He et al. [69] showed that inhibiting the chloride co-transporter (NKCC1) with bumetanide treatment during an early developmental period normalizes the development of thalamocortical synapses in the mouse somatosensory cortex. Early inhibition of NKCC1 was sufficient to reduce the size of whisker evoked activity spread in the adult mouse cortex. These studies suggest that early treatment leave a long lasting impact on brain and behavior in *Fmr1* KO mice. Hodges et al. [71] showed that a single seizure in *Fmr1* KO mice during early development can lead to long lasting impairments in cognitive behaviors. Approximately 20–30% of children with FXS exhibit seizures. The potential impact of early life seizures on adult treatment efficacy is unknown in FXS. A systematic evaluation of treatment age and duration of drug efficacy on a number of symptoms is, therefore, a critical need in FXS research and more generally in autism spectrum disorders.

### To what extent is developmental compensatory plasticity involved in adult pathophysiology?

An intriguing finding across studies is the consistent observation that many of the phenotypes are either seen only transiently, or show considerable changes in direction and/or strength during development. This seems to be the rule rather than an exception and point to the clear importance of studying developmental trajectories to understand mechanisms of pathophysiology and to identify optimal treatment points. The topic of fluctuating or transient phenotypes has been reviewed thoroughly by Meredith et al. [102] and Meredith [103]. Additional studies since then continue to show similar trends. For example, when sound driven single unit spiking responses are evaluated in developing and adult auditory cortex, hyper-responsiveness is observed at P21 in adults, but not in P14 and P30, in *Fmr1* KO mice [140]. Hyper-responsivity develops between P14 and P21. A study that examined mouse cortical EEG responses at P21, P30, and adults came to a similar conclusion [142]. The sound-driven non-phase-locked gamma power of EEG responses was significantly elevated in the auditory cortex and frontal cortex of P21 and adult *Fmr1* KO mice compared with WT. However, at P30, the non-phase-locked power was reduced in the Fmr1 KO mice, again pointing to developmentally fluctuating phenotypes. The resting gamma power was elevated in the Fmr1 KO mouse frontal cortex at all ages tested pointing to region specific developmental trajectories of different phenotypes.

Wen et al. [141] suggested that the hyperexcitability at P21 may arise due to impaired function of parvalbumin (PV) positive inhibitory neurons due to the altered expression of specialized extracellular matrix assemblies called perineuronal nets (PNN). The altered function of PV cells is also seen during development in the somatosensory cortex [107] and adult visual cortex [48]. PNNs protect PV cells against oxidative stress and also function to increase excitability of these cells. Reduced excitability of PV cells can lead to reduced inhibition in the circuit and hyperexcitability. Abnormal development and dysfunction of GABA neurons is also seen as a key mechanism underlying other neurodevelopmental disorders including Rett [23] and Angelman syndrome. In Rett syndrome model mice, there is an abnormally early maturation of PV neurons in the visual cortex [32]. Notably, daily low-dose Ketamine treatment of Mecp2 mice can reverse symptoms by modulating NMDA receptors on PV neurons [111]. The treatment that started at ~P30 (onset of regression) was as effective as the treatment that started earlier in development, consistent with findings of Guy et al. [55].

PNNs around PV neurons can be modified by actions of an endopeptidase, matrix metalloprotease-9 (MMP-9), a target of FMRP. In the KO mice, the lack of FMRP leads to increased MMP-9 levels throughout the development and deterioration of PNN. The P14–P21 developmental window is a time of considerable experience dependent plasticity in the mouse auditory system. Abnormal response during this critical period plasticity window is predicted to have consequences for the development of auditory processing [83]. By P30, however, PV/PNN expression and response magnitudes are normal in the KO mouse auditory cortex consistent with other findings regarding transient phenotypes. In the adult brain as well, PV/PNN expression is normal, but hyper-responsivity is present suggesting potentially novel mechanisms in adulthood, or a “sleeper” effect that arises as a result of developmental alterations. Exposure of developing *Fmr1* KO mice between P5 and P21 to sounds reduces neural correlates of auditory hypersensitivity including a normalization of ERP responses, dendritic spine density, and PV neuron density [87] suggesting the potential use of non-pharmacological treatment strategies either in isolation or in combination with drugs. Together, these studies call for early treatment to observe if adult effects can be prevented. If transient changes in early development can be prevented, does that have long-term beneficial effects in disease progression is a question that has received very little attention in the field.

## Human development and FXS

Similar issues emerge when examining human data across the lifespan. FMRP is expressed across cell types in multiple brain regions; we have little to no understanding of changes in FMRP expression across development, and it becomes even more difficult to disentangle the effects of genetic mutation from the effects of altered developmental experience in the human brain. FMRP influences synaptic plasticity (the “activation” effect) as well as the structural integrity (“organizational”) of the brain. How this translates to phenotypic changes and which aspects are most critical for successful intervention are unknown. There is a paucity of human work that links neural (dys) function to both disease phenotype and molecular etiology in a developmental framework. This connection is vital for patient stratification and evaluation of potential biomarkers in future treatment studies in order to optimize outcome measures to correlate with clinically meaningful symptomatic change.

Animal studies have demonstrated the developmental regulation of FMRP expression in neural tissue. In adults, FMRP is widely expressed in epithelial cells, neurons, and glia [11, 30, 47]. We have limited knowledge regarding FMRP expression across development in the human brain. FMRP expression is seen in fetal neural tissue as early as 13 weeks gestation [1], but little to no FMRP expression was noted in a 3- to 7-week embryo [2]. How FMRP expression evolves throughout fetal and early development in humans is unknown. FXS is often not diagnosed until the toddler years; therefore, studies in very young children with FXS are limited but suggest that detectable phenotypic changes occur quite early. Behavioral [119] and neural [133] changes have been identified as early as 6 months old. However, studies have only examined children down to age 5 months, and longitudinal data is lacking making our understanding regarding the stability of neural and symptomatic changes in FXS limited. We have some information about the relationship between neuroendophenotypes and FMRP levels. Some have probed this question directly by measuring FMRP. Others studies have stratified participants according to sex, mosaicism, and methylation as a proxy for direct FMRP measurements. The studies outlined below suggest complex developmental trajectories across a number of phenotypes in FXS and highlight the need to examine developmental trajectories rather than single time points in relation to molecular changes.

### The brain in FXS

Preclinical work suggests wide reaching effects of FMRP on neural structure and function. Human neuropathologic studies demonstrate dendritic spine abnormalities in FXS [78–80] with limited consistent gross pathologic change [1, 51, 121]. Neurophysiologic studies, on the other hand, do show some replicated changes in neural structure and function in FXS across the lifespan.

The earliest changes in the FXS brain to date have been seen in the white matter at 6 months old using diffusion tensor imaging (DTI) [133]. In their study of 27 infants (22 males) with FXS and neurotypical controls (*n* = 73) followed from 6 to 24 months, Swanson et al. [108] found lower fractional anisotropy (FA) across long-range pathways within the brain: those connecting subcortical structures (including the thalamus, basal ganglia, and cerebellum) to the prefrontal cortex, the corpus callosum in areas linking the primary and premotor cortices and in the bilateral uncinate fasciculus. Authors found that the lower FA compared with controls was stable from 6 to 24 months of age and was strongly correlated to IQ in the FXS group. Studies in older individuals with FXS found increased FA in regions that overlap somewhat with regions identified to have decreased FA in the infant work. Green et al. [52] found increased FA in the inferior longitudinal, inferior frontotemporal, and uncinate fasciculi in a group of 40 young adults with FXS (25 female). Hall et al. [59] had very similar results. In contrast, Barnea-Goraly et al. [10] found decreased FA in a small group (*n* = 10) of females (ages 13.1–22.7) with FXS in the frontal-striatal WM, with a small region of increased FA in the superior temporal gyrus. The small number within this study along with the participants being all female may help to explain these differing results. There is also the potential that the regional developmental regulation of FMRP is different in different neural systems which can lead to divergent effects in the adult brain. The discrepancy seen between the infant study and later finding of increased FA in overlapping regions is consistent with a putative role for FMRP in white matter maturation. As outlined above, FMRP regulates the dendritic translation of a wide variety of proteins critical for synaptic and dendritic function [125]. Dysfunction in this system has a potential effect on pruning and resultant modeling of neural systems. In addition, FMRP has a putative role in the regulation of myelin basic protein (MBP) [47, 110], which plays an important role in the developmental regulation of myelination [13]. Recent work in the mouse model has demonstrated delayed myelination in early development [110].

Neuroimaging studies have suggested a central role for frontal-striatal circuitry in the neuropathology of FXS. Enlargement of the caudate nucleus is one of the most consistent findings in the FXS neuroimaging literature [62, 72, 74, 144] often in association with abnormalities in frontal lobe volume [14, 17, 18, 62]. Caudate abnormalities are seen across MRI modalities, in multiple age groups and in cross-sectional and longitudinal design (e.g., [20, 49, 50, 90]). Haas et al. [56] demonstrated structural changes in frontal-striatal circuits in toddlers with FXS. In a functional imaging study examining frontal-striatal functioning utilizing a go/no go task, Hoeft et al. [73] demonstrated frontal-striatal changes mediated by FMRP and striatal dysfunction. Some studies indicate a more striking increase in males with FXS [35, 49, 50] which may indicate a direct relationship between FMRP expression and caudate structure; however, others have not seen this sex difference [90]. Studies exploring the relationship between FMRP levels and caudate structure more directly support a relationship. Hoeft et al. [74] demonstrated a negative correlation between caudate volume and FMRP in young children with FXS. Gothelf et al. [49] found the same relationship in an older group of participants. FMRP was significantly negatively correlated with caudate volume. Gothelf and colleagues also found FMRP levels to be related to the increased volume of the posterior vermis of the cerebellum, while Hoeft et al. [74] did not see this same relationship in early childhood. Overall, the increased volume of the caudate nucleus appears to be associated with less FMRP and more severe phenotypic expression across different behavioral domains [120, 144]. Stereotypic behavior and inappropriate speech have each been linked to caudate structure in FXS [112, 144]. This is one of the few areas where we start to see a potential molecular-brain-symptom connection emerging that is present in early development emerging in the literature.

We see changes in the striatum in the few longitudinal neuroimaging studies of young children with FXS as well. In a study including 53 male toddlers (18 to 42 months of age) with FXS [68], there was a stable increase in the volume of the caudate, putamen, and globus pallidus, most striking in the caudate nucleus. Bruno et al. [19] used both cross-sectional and longitudinal approaches to distinguish two groups (*N* = 13 and *N* = 24) of boys (mean age 2.89 years at baseline; mean age = 4.9 years at follow-up) with FXS differentiated by enlarged gray and white matter regions including the caudate, thalamus, frontal lobe, temporal lobe, and cerebellum. The boys with FXS and larger brain volume (*N* = 13) showed reduced adaptive behavior, lower initial IQ, and more ASD symptoms than the comparison FXS group of boys (*N* = 24). Though this group generally had a more severe phenotypic profile, there was no correlation found with FMRP in this cohort.

Additional regions consistently linked to FXS, where changes have been documented early in development, include the amygdala [67, 74, 82] and cerebellum [51, 54, 74, 105, 117, 143]. Each of these structures has been linked to specific features of the FXS phenotype [61, 138]. However, characterization of the influence these brain regions have on the FXS phenotype through development is lacking.

A preliminarily look at the FXS brain from a network perspective in older individuals with FXS [17, 18] suggests that there is limited connection between subcortical and cortical systems. Bruno and colleagues [18] found that the caudate and amygdala were less involved in large scale neural networks in FXS. The result of changes in subcortical structures may be a decreased ability to recruit higher order brain regions to modulate behavior.

EEG studies in adolescents and adults with FXS have demonstrated a robust and reliable phenotype of altered gamma band activity [37, 38, 137] that is replicated in the mouse model [99] and correlates with clinical symptoms including sensory hypersensitivities and social/behavioral measures [36–38, 137]. None of these studies have examined the relationship between EEG results and FMRP. When looked at indirectly by comparing males and females, most have seen no difference in these groups, with one exception [36]. There have been no studies examining this phenotype in early development or longitudinally. Given the developmental changes seen in EEG phenotypes with the mouse model, and the central role EEG plays in translational studies of FXS (discussed in more detail below), this will be an important question to address in the near future.

### Behavioral and cognitive development

Standardized cognitive and adaptive measures show changes prior to age one. Roberts et al. [119] demonstrated differences as early as 6 months old when examining cross-sectional profiles on the Mullen Scales of Early Learning (MSEL, [106]). Previous studies had demonstrated changes as early as 9 months old in infants with FXS for both cognitive and adaptive measures [21, 118]. Overall, studies examining adaptive and cognitive functioning in FXS suggest a delay followed by steady gain of skills and then what may appear to be a decline, but is more likely a plateau of skills compared with the normative sample used to calculate standard scores [21, 33, 40, 85, 118, 130, 131]. This apparent decline is less severe in females, believed to be due to the expression of some FMRP in females due to their necessary mosaicism [85, 145]. Research directly examining FMRP expression and cognitive behavioral phenotypes in FXS is quite variable. Many have linked FMRP to general cognitive impairments in FXS [7, 8, 96, 97, 130, 134] while others show no relationship [66, 130].

There is some evidence that this pattern in skill development is not uniform across cognitive domains. For instance, Quintin et al. [115] followed cognitive development in 114 males and 70 females with FXS evaluated first at a mean age of 11.3 years with 1–6 years between follow-up points with up to 3 cognitive measures using the Wechsler Intelligence Scale for Children [139]. Over time, a widening of the gap in FXS compared with the normative IQ sample was noted in verbal comprehension, perceptual organization, and processing speed with a narrowing of the gap noted in freedom from distractibility. In a small 1 year follow-up study focused on attention and working memory in FXS, 21 boys with FXS and an equal number of matched typically developing youth (mean age = 8.8 years at first visit), attentional control and working memory were noted to improve over time in the FXS group mirroring developmental change in the control group [25]. This is in contrast to other studies that have demonstrated attention skills similar to mental age matched controls [132] or a relative impairment [123]. There is some limited evidence deficits in FMRP are specifically linked to executive dysfunction in FXS [41, 88, 95, 98], most consistently with working memory and attention. However, some phenotypic studies suggest sparing in this domain [25, 115] which is at odds with this interpretation.

Longitudinal profiles of language development are limited in FXS and predominantly in older individuals. One recent study [116] examined very early language in a small sample of infants and toddlers with FXS using an automated vocal analysis system. Results in this group of 11 males with FXS (ages 17 to 64 months) showed that infants and toddlers with FXS had significantly less vocalizations per hour than typically developing peers, but similar numbers to developmentally age-matched peers suggesting that language in this age group is delayed in proportion to general developmental delay. There was also significant variability in the FXS group in this small study. Martin et al. [101] followed 29 youth with FXS without ASD, 40 youth with FXS and ASD, 34 youth with Down syndrome, and 40 typically developing mental age matched youth ages 3.1 to 16.0 years over a period of 3 years. In these samples, the rate of language gain was higher in the typically developing group compared with those with FXS regardless of ASD status showing a similar language development trajectory to those with Down syndrome. Together, this research suggests that language development is tightly linked to overall cognitive development in FXS and appears to follow a similar developmental trajectory.

There are mixed results when examining the emergence and development of ASD symptoms over time in FXS [6–9, 42, 65, 91, 122]. However, the majority of these studies have been limited by small sample size and/or cross-sectional design. Two larger longitudinal studies [65, 91] suggest that autism symptoms in FXS may increase with age with the most prominent increase noted in social communication features consistent with ASD. Overall, we see that those children with FXS and comorbid ASD have a more severe presentation. For example, Caravella et al. [21] examined the development of adaptive skills in relation to autism symptoms in male infants with FXS (*N* = 25) compared with typical developing male infants (*N* = 24) and infant male siblings of persons with ASD (*N* = 27) [21]. They found that increased severity of ASD symptoms was related to increased deficit in adaptive skills at 24 months of age. We see a similar pattern of results with ASD symptoms in relation to FMRP. Some have demonstrated a relationship between peripheral FMRP levels and ASD symptoms [65, 70]. Hatton et al. [65] examined the role of FMRP in the expression of ASD symptoms and found a significant relationship with increased ASD symptoms related to lower FMRP levels. Hessl et al. [70] also found a link between FMRP and ASD symptoms, but only in girls. Other studies show no link [7, 8, 63, 94].

In terms of interfering behaviors, Hustyi et al. [76] examined the longitudinal trajectory of interfering behavior in 64 males and 60 females with FXS (ages 2 to 26 years) using two more assessments with the Aberrant Behavior Checklist (ABC [3];) with an average interval of 4 years between evaluations with a mean subject age of 11.6 years at first assessment [76]. In this cohort, irritability (aggression, self-injury, and severe tantrums) and hyperactivity significantly reduced with age and social withdrawal, stereotypic behavior, and inappropriate speech did not change over time as measured by the ABC. This is similar to earlier longitudinal studies examining interfering behaviors [34, 64]. Hall et al. [58] found no relationship between interfering behaviors and FMRP. All studies have examined a rather large age range. As a result, we have some understanding of improvement and stability of symptoms in later childhood, but we have little information about the emergence of interfering behaviors and early modifying factors.

All behavioral phenotypic studies outlined above have similar limitations. Most have small sample sizes and limited time for follow-up. Symptom clusters are often examined in isolation, and there is little exploration of the relationship between phenotypic changes and brain development or FMRP levels.

### What about FMRP?

The lack of FMRP is the core deficit of FXS. As noted above, FMRP levels have been linked to a small group of symptoms and neural changes in human studies of FXS; however, this work is limited in sample size and reproducibility. FMRP is difficult to measure, and there are currently many methods used for FMRP quantification with no general consensus regarding the optimal method (reviewed in [89]). As a result, the studies above have relied on a variety of measures making comparison across studies more challenging. There have been no large studies examining test-retest reliability, inter- or intra-individual variability of FMRP measurement in individuals with FXS. Until this hurdle has been overcome, future studies probing the link between molecular and phenotypic changes in FXS will be at a significant disadvantage.

### Summary of human data

Human data suggest that very early in life brain deficits exist in FXS and can be captured clinically as well as neurologically. In each domain of development (behavior, cognition, language), limited longitudinal data exists in FXS. Despite the limited nature of the data, the available body of literature does suggest delayed but steady improvement in cognition, adaptive behavior, and language in those with FXS without ASD. In addition, FXS + ASD likely leads to a more severe presentation overall in interfering behaviors and in social-communication deficits. We have little data to link specific brain abnormalities to these phenotypic profiles. Interpretation of these results is impeded by diverse methodology in following subjects with FXS, scarce long-term longitudinal data, and small sample sizes. In addition, this research suggests there are some deficits and behaviors more related to cognitive impairment and global delay, while other changes may be independent of intellectual disability in FXS such as attention and anxiety, though there has been very limited work in young children examining these symptoms in a systematic manner.

It will be important to longitudinally examine both the phenotypic characteristics in FXS, changes in neural structure and function, and changes in FMRP and how they relate. Disentangling the influence of cognitive impairment and global delay will require the inclusion of developmentally matched control groups.

## Challenges to integrating human and animal model work

An important challenge in translational neuroscience is the identification and validation of conserved biomarkers across species that can facilitate the treatment development pipeline. This is even more difficult when comparing phenotypes at different developmental ages, because there is no good way to identify equivalent ages across species. Sensory system development may provide an opportunity to effectively address this challenge for multiple reasons. Sensory (particularly auditory) hypersensitivity is present in both humans with FXS and in the *Fmr1* KO mice. There is a rich literature on normal sensory system development in both humans and animals, including mice. This includes a number of studies on the long-lasting effects of neural and behavioral manipulations during critical period plasticity windows, and how these windows can be changed. Equivalent developmental periods (irrespective of chronological age) of increased brain sensitivity to sensory manipulations can be established across species. The brain circuits that shape low-level sensory processing are likely to be more conserved across species compared with circuits involved in complex cognitive and social functions. There is relatively less information on the development of circuits involved in cognitive and social behaviors. Taken together, these considerations suggest that studies of sensory system development offer an avenue to integrate human and mouse work.

Recent studies that were inspired by the above considerations used EEG/ERP recordings and coordinated experimental design to identify remarkably similar auditory response phenotypes in humans with FXS [36–38, 137] and the *Fmr1* KO mice [99, 100, 129, 142]. EEG recordings show that in both species there is increased baseline gamma band power, reduced inter-trial coherence of sound evoked oscillations, enhanced N1 component amplitude of ERP, and reduced habituation of auditory ERP amplitudes. Gamma band abnormalities are correlated with parent report of sensory hypersensitivity and social communication deficits, and a number of clinical ratings in humans, and are sensitive to drug treatment in humans [124] and in mice [129]. In mice, these response abnormalities are present from P21 [142] and may develop between P14 and P21 due to abnormal maturation of PV expressing GABA neurons [141]. Together, these studies suggest EEG/ERP phenotypes can be used as biomarkers for stratification and drug-outcome prediction in humans and to understand pathophysiological mechanisms and drug development in mice.

Despite the wealth of data on adult human EEG/ERP data in FXS, relatively few studies have examined developmental changes in EEGs in children. The lack of longitudinal and early EEG data is a significant gap in the literature. Therefore, a number of fundamental questions regarding EEG abnormalities in FXS remain unanswered. In neurotypical children, gamma oscillations are detected around 16 months of age and continue to increase until approximately age 5 [135]. During those first 2 years of neurotypical growth, we see significant within network synchronization across functional networks [43]. The emergence of cortical networks and synchronized oscillations are linked. The increased precision with which oscillations are synchronized likely relates to the maturation of the organization—both structurally and functionally—in cortical networks. The emergence of synchronized gamma oscillations is linked to specific cognitive skills, for instance perceptual binding and early gamma changes are related to the later development of cognitive and language skills [43]. Early longitudinal EEG studies are needed in order to understand how the emergence of frequency bands may be altered in FXS and how changes are linked to symptoms. Multimodal studies that incorporate multiple objective measures are required to expand our mechanistic understanding of FXS. Developmental profiles of EEG/ERP changes may be a useful marker to test the outcomes of early versus late treatments in humans. The use of analogous stimulus design and recording methods in mice and humans may serve as a paradigm to identify objective outcome measures specific for different developmental time points. But such studies are currently lacking.

In addition, we need a better understanding of the changes across modalities probing brain function and how these changes relate to both the underlying FMRP deficit and resulting phenotypic presentation. MRI studies have revealed early changes in the FXS brain and suggest that imaging based biomarkers can be used to test the outcomes of early interventions. However, no studies have examined the link between EEG abnormalities and localized brain changes identified in MRI studies. Combining neuroimaging and EEG across development may offer important clues regarding the localization of change in the FXS brain. In FXS, we see abnormalities in functional networks in adolescents [60] but have no understanding regarding when these changes emerge. The amplitude of BOLD signals in functional MRI is closely and positively correlated with the entrainment of neurons into synchronized gamma band oscillations. Therefore, we should see the coincident emergence of functional networks and gamma synchronization across development. In addition, brain changes in both humans and mice should be examined in relation to FMRP levels and phenotypic presentation to disentangle the effect of general developmental delay from specific effects of FXS.

In mice, the relationships between physiological responses and perception should be examined in more detail. Goel et al. [48] showed that reduced neural visual orientation selectivity in *Fmr1* KO mice was related to poor behavioral orientation selectivity. This was shown to be related to abnormal PV cells function, because increasing PV cell activity reduced both neural and perceptual deficits in the KO mice. Interestingly, similar behavioral deficits were also seen in humans with FXS, demonstrating a path towards integrating human and mouse work. In humans, the use of more quantitative and objective measures rather than parent report will add to the richness of phenotypic data and likely lead to a more complete understanding of brain-behavior relationships in FXS. This includes measures such as expressive language sampling, eye tracking, and investigator scored social measures such as the BOSCC (brief observational measure of social communication, [84]). A better understanding of these early brain-behavior changes has implications in how we approach treatment development in FXS.

Multiple lines of evidence in the *Fmr1* KO mouse indicate abnormal GABAergic inhibition across different brain areas. In humans, only a few studies have examined inhibitory mechanisms. D’Hulst et al. [26] showed reduced GABAa receptor binding using positron emission tomography (PET) scan. Morin-Parent et al. [104] used transcranial magnetic stimulation (TMS) to reveal abnormal functional inhibition in humans with FXS. As seen with mice, these results point to reduced inhibition as a mechanism of hyperexcitability. The human studies were done in adolescents and/or adults. The timing of neural changes can offer insight regarding critical time points for intervention in this system. For instance, in early development, GABA is depolarizing to neurons and then later become hyperpolarizing. Recent work suggests a delay in the GABA reversal is delayed in FXS [69]. This may be linked to changes in plasticity in early development that we may be able to capture. However, this may also be a nonspecific compensatory downstream effect of the FMRP deficit. Delay in GABA reversal is seen in models of other genetic syndromes with intellectual disability, most notably Down syndrome [28, 77]. Early compensatory changes in GABAergic neurotransmission may be related to later changes in the excitatory-inhibitory balance seen in FXS and other related disorders [4]. This E/I imbalance is thought to drive much of the current EEG data, making this perhaps a more nonspecific marker in the population rather than specific to the FXS disease state. Combining EEG, MRI, and TMS may help disentangle changes in excitatory and inhibitory processes in children with FXS [22, 113]. Early studies, like those outlined above with appropriate control groups, are needed to tease these questions apart.

## Conclusions and future experiments

While some work has been done to describe the developmental trajectory of FXS in humans, many questions remain. First, limited functional and structural serial brain imaging data is available, and the data is limited to date within the youngest children. Second, while electrophysiological abnormalities have been well characterized in humans with FXS, no developmental or long-term EEG follow-up studies have been conducted in FXS. Third, phenotypic characterization has been limited to primarily standardized measures and have not examined important aspect of the phenotype, specifically anxiety, attention, and sensory dysfunction in depth during early development. These shortcomings will inhibit targeted treatment work in the field because without developmental understanding of brain change over time in FXS it is difficult to evaluate the target engagement of a particular drug or therapy or combination of drug + therapy at specific windows of development. This is of particular importance if a treatment is being administered over time to potentially improve the course of development in FXS and given the advancements in the treatment development field looking at long-term drug + non-drug intervention as a means to enhance developmental gains. Specifically, the FX-LEARN project evaluating the effects of the selective metabotropic glutamate receptor type 5 (mGluR5) antagonist on language learning in young children with FXS (NCT02920892) is an example of a targeted treatment over a long time period that would significantly benefit from a comprehensive foundation of developmental understanding in FXS.

The lack of a firm developmental understanding of the presentation and brain biology of FXS and how that relates to cognition and behaviors impairs clinicians’ ability to discuss with families and caregivers what can be expected over time. These deficits are being addressed in part by the Fragile X FORWARD project creating a registry and clinical database describing the natural history of FXS [126]. Despite these efforts, the field still lacks more biologically based supported effort to track developmental change including a lack of understanding of potential change in FMRP expression or brain physiology over time. Given these challenges, new targeted treatment approaches that may seek to enhance FMRP expression [45, 57, 92, 146] will be challenged to define effective target engagement with brain pathophysiology at a specific stage of development. One of the biggest gaps in the literature on FXS is worth reiterating here. Whether early developmental treatment of FXS has long lasting benefits is unclear. In addition, how combinations of treatment either at the same time or staggered at appropriate time points have long lasting benefits has not been tested. Whether reinstatement of FMRP in adults will be useful in reducing symptoms will depend critically on whether FMRP plays an irreversible brain organizational role during early developmental critical periods. Studies that address these critical period questions, like those done in Angelman and Rett syndromes, are of immediate need in *Fmr1* KO mice.

## Data Availability

Not applicable.

## Abbreviations

ABC: Aberrant Behavior Checklist
AS: Angelman syndrome
ASD: Autism spectrum disorder
EEG: Electroencephalography
ERP: Event-related potential
FMRP: Fragile X mental retardation protein
FXS: Fragile X syndrome
MRI: Magnetic resonance imaging
P: Post-natal
PET: Positron emission tomography
PNN: Perineuronal nets
PV: Parvalbumin
sLNv: Small ventrolateral neurons
TMS: Transcranial magnetic stimulation
